# Visual field defects and retinal nerve fiber imaging in patients with obstructive sleep apnea syndrome and in healthy controls

**DOI:** 10.1186/s12886-018-0728-z

**Published:** 2018-03-02

**Authors:** Paula Casas, Francisco J. Ascaso, Eugenio Vicente, Gloria Tejero-Garcés, María I. Adiego, José A. Cristóbal

**Affiliations:** 1 Department of Ophthalmology, Hospital Clínico Universitario “Lozano Blesa”, San Juan Bosco 15, ES-50009 Zaragoza, Spain; 2 Department of Otolaryngology, Hospital Universitario “Miguel Servet”, Zaragoza, Spain; 3 Instituto de Investigación Sanitaria Aragón (IIS Aragón), Zaragoza, Spain

**Keywords:** Visual field, Automated perimetry exam, Optical coherence tomography, Obstructive sleep apnea syndrome, OSAHS

## Abstract

**Background:**

To assess the retinal sensitivity in obstructive sleep apnea hypopnea syndrome (OSAHS) patients evaluated with standard automated perimetry (SAP). And to correlate the functional SAP results with structural parameters obtained with optical coherence tomography (OCT).

**Methods:**

This prospective, observational, case-control study consisted of 63 eyes of 63 OSAHS patients (mean age 51.7 ± 12.7 years, best corrected visual acuity ≥20/25, refractive error less than three spherical or two cylindrical diopters, and intraocular pressure < 21 mmHg) who were enrolled and compared with 38 eyes of 38 age-matched controls. Peripapillary retinal nerve fiber layer (RNFL) thickness was measured by Stratus OCT and SAP sensitivities and indices were explored with Humphrey Field Analyzer perimeter. Correlations between functional and structural parameters were calculated, as well as the relationship between ophthalmologic and systemic indices in OSAHS patients.

**Results:**

OSAHS patients showed a significant reduction of the sensitivity for superior visual field division (*p* = 0.034, t-student test). When dividing the OSAHS group in accordance with the severity of the disease, nasal peripapillary RNFL thickness was significantly lower in severe OSAHS than that in controls and mild-moderate cases (*p* = 0.031 and *p* = 0.016 respectively, Mann-Whitney U test). There were no differences between groups for SAP parameters.

We found no correlation between structural and functional variables. The central visual field sensitivity of the SAP revealed a poor Pearson correlation with the apnea-hipopnea index (0.284, *p* = 0.024).

**Conclusions:**

Retinal sensitivity show minor differences between healthy subjects and OSAHS. Functional deterioration in OSAHS patients is not easy to demonstrate with visual field examination.

## Background

Obstructive sleep apnea-hypopnea syndrome (OSAHS) is a disorder characterized by brief episodes of complete or partial upper airway collapse during sleep. When those apnea-hypopnea events sum five or more events per hour, a pathological breathing status appears. Numerous ophthalmological disorders seem to be associated with OSAHS, including floppy-eyelid syndrome or central serous chorioretinopathy. Moreover, some authors suggest that certain optic nerve (ON) disorders such as papilledema, glaucoma or non-arteritic anterior ischemic optic neuropathy showed an increased incidence in the obstructive disease [1–10].

Changes in the retinal nerve fiber layer (RNFL) thickness have been reported in OSAHS by multiple authors. These alterations appear even in individuals in whom glaucomatous neuropathy has been ruled out, proposing therefore the breathing disease could be “per se” an aggressive agent for the ON [11–16].

Studies describing the progression of the most common neuropathy in our specialty, glaucomatous neuropathy, have contributed to establish a relationship between the functional visual impairment and the structural damage of the ON [17, 18]. Other pathologies, as sclerosis multiple, also seem to have a good agreement and correlation between abnormalities detected by standard automated perimetry (SAP) and RNFL measurements, as Cheng et al. found in eyes with optic neuritis secondary to this cause [19].

The aim of this study was to investigate whether there is a visual field (VF) functional deficit in OSAHS patients compared to healthy individuals. And to study if there is a correlation between functional variables and structural (OCT) variables in OSAHS.

## Methods

Eighty OSAHS patients were consecutively recruited in the Otolaryngology Department at the Hospital Miguel Servet, in Zaragoza, Spain. All patients had a newly discovered and previously untreated mild to severe OSAHS according to clinical features and apnea–hypopnea index (AHI) greater than 4. Before OSAHS was confirmed, patients completed a questionnaire concerning epidemiological data and information about symptoms such as loud snoring, observed apnea, or excessive daytime sleepiness. The most common vascular risk factors were studied and treated if necessary.

Patients were subsequently referred for an ophthalmological examination to the Ophthalmology Department at the Hospital Clínico Lozano Blesa in Zaragoza, Spain, between December 2010 and March 2012. Patients with history of stroke with central apnea, chronic uveitis, antiglaucomatous drug usage, optic neuropathy, ocular trauma or surgeries were excluded from this study. After appropriate information, written informed consent of all subjects was obtained. The research followed the tenets of the Declaration of Helsinki, and the protocol was approved by the local Ethics Committee.

The control group included 40 age-matched healthy subjects, who were recruited among relatives and employees at the Hospital Lozano Blesa. Selection was made with Berlin questionnaire (evaluating the functional signs of OSAHS, 86% sensitivity for diagnosis and 77% specificity) [20, 21]. Epidemiological data were collected and smoking habit and vascular risk factors were treated in the same way.

All OSAHS and controls underwent a complete ophthalmologic examination, including best-corrected visual acuity (BCVA), ocular motility, slit-lamp biomicroscopy, Goldmann applanation tonometry, gonioscopy, Humphrey automated VF, and OCT examination. Values of the right eyes were selected for analysis except when they did not fulfill the inclusion criteria; in this case, left eyes were selected.

At least two reliable SAP were performed to minimize the learning effect. VFs were evaluated with a Humphrey Field Analyzer perimeter model 740i (Zeiss Humphrey Systems, Dublin, CA) by using the 24–2 SITA fast strategy, with a Goldman size III stimulus on a 31.5-apostilb background. Near addition was added to the subject’s refractive correction. If fixation losses were higher than 20% or false positive or false-negative rates were higher than 15%, the test was repeated. Each perimetry was performed on different days to avoid the fatigue effect and the same experienced examiner conducted all scans.

OCT was performed with the Stratus OCT (Carl Zeiss Meditec, Dublin, CA, USA) following 1% tropicamide instillation. Only high-quality images were included. Each patient underwent scans to measure peripapillary RNFL thickness, which was automatically calculated by the fast RNFL algorithm. We considered the overall thickness and per quadrant.

Examiners were masked to the diagnosis. All participants had a BCVA of 20/30 or better, with a refractive error lower than three spherical or two cylindrical diopters. Intraocular pressure (IOP) > 21 mmHg, posterior segment pathology or patients with media opacification were excluded. Regarding VF, eyes with defects compatible with glaucoma (nasal step, paracentral or arcuate scotomas, or arcuate blind spot enlargement) with a pattern standard deviation (PSD) significantly elevated beyond the 5% level and/or a Glaucoma Hemifield Test outside normal limits were also excluded.

**Fig. 1:**
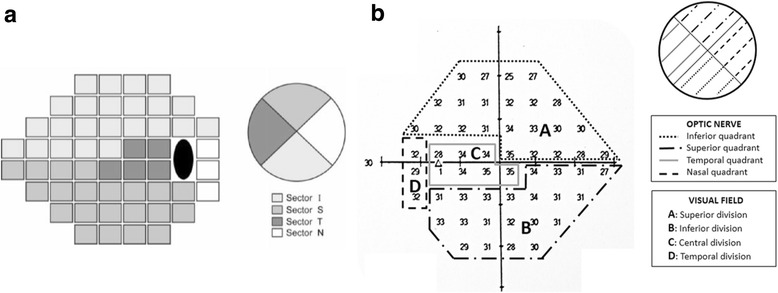
Corresponding areas: visual field – optic nerve. **a** Cheng et al. map. Correlation between OCT RNFL quadrants and corresponding sectors on SAP 24–2. **b** Topographical division on map of absolute retinal sensitivity used in our study, based on the topographic map proposed by Cheng et al. Note the inclusion of the points corresponding to the blind spot in the central division average sensitivity calculations

For comparison we used Humphrey global indices such as mean deviation (MD) and PSD, and we also calculated mean sensitivity (MS) recording each of the threshold values in decibel scale. Sector MD was calculated by averaging the deviation values on total-deviation plots for each sector. To investigate correspondence between structure and function, we used a more simplified version of the topographic map obtained by Garway-Heath [17], proposed by Cheng et al. [19]. Nevertheless, we introduced some subtle variations on Cheng’s map, like the use of all sensitivity points, including the blind spot ones (Fig. 1).

### Statistical analysis

Data analysis was performed by using Statistical Package for Social Sciences software (version 19.0, Chicago, IL, USA). Values were presented as mean ± standard deviation, and expressed in microns for the peripapillary RNFL thickness and in decibels for the VF sensitivities. Qualitative differences between the studied variables were assessed by using Pearson’s chi-squared test.

Differences between controls and OSAHS were tested by Student’s-t test when normality and equality of variances were proved. If these conditions were not satisfied, the non-parametric Mann-Whitney U test was used.

In a second analysis, OSAHS sample was divided according to severity into two groups: those with mild-moderate OSAHS (group 1, AHI ≥5 and < 30) and those with severe OSAHS (group 2, AHI ≥30). Both groups were compared to controls. Quantitative differences between the three groups were compared by using one-way ANOVA test, once normality and homogeneity was proved with Shapiro-Wilk and Levene’s tests. In the event of breach of the homogeneity and normality assumption, the non-parametric Kruskal-Wallis H test was applied. In those cases where differences had been statistically significant, two by two Scheffé comparison (after ANOVA test) or Mann-Whitney U analysis (after Kruskal-Wallis H test) were performed to know which groups were different.

The relationship between structural and functional variables were analyzed by applying linear, logarithmic, inverse, quadratic and cubic models in OSAHS and in controls separately. In addition, Pearson’s correlation coefficient between AHI and VF indices in OSAHS were evaluated. A *p* value < 0.05 was considered statistically significant.

## Results

Of 80 consecutive OSAHS who accepted to participate, 63 patients (66.9%) were included in the study (51 right eyes and 12 left eyes), whereas 36 patients (33.1%) were excluded. Twenty-nine patients had a mild-moderate OSAHS (46%), and the other 34 patients (54%) had a severe disorder.

In the control group, 40 individuals classified as “low risk subjects” by the Berlin questionnaire were examined. Two patients were excluded after exclusion criteria application, and finally the group was composed of 37 right eyes and one left eye of 38 healthy subjects.

Age showed no statistically significant difference between groups, neither when dividing the OSAHS group according to its severity. In OSAHS group, although more men than women were enrolled, we did not consider this difference because gender has no effect on RNFL thickness as previously mentioned [22]. Body mass index was not matched, and was higher in OSAHS than that in controls (*p* < 0.001). Nevertheless, no significant differences in vascular risk factors (hypertension, diabetes and dyslipidemia) and prevalence of smoking habit between cases and controls were found.

Table 1 shows the results of IOP, peripapillary RNFL thickness and VF parameters in controls and OSAHS. Differences between groups were just found in the VF superior division, sensitivity was higher in healthy subjects than in OSAHS ones. MD showed results close to the statistical significance.

**Table 1:** Comparison of IOP, RNFL thickness and Humphrey VF parameters between controls and OSAHS patients

Variable	CONTROLS (*N* = 38)	OSAHS (*N* = 63)	*p*-value
IOP	15.1 ± 2.1 [10–19]	15.7 ± 2.8 [10–22]	0.218^*^
Average RNFL	98.60 ± 10.95 [68.86–117.54]	98.79 ± 10.55 [76–125.33]	0.932^*^
RNFL superior Quadrant	119.89 ± 19.32 [69–159]	123.17 ± 16.35 [85–162]	0.385^*^
RNFL nasal Quadrant	80.08 ± 16.92 [52–126]	74.86^†^ ± 15.86 [47–117]	0.128^*^
RNFL inferior Quadrant	124.21 ± 13.71 [93–160]	123.84 ± 17.29 [91–166]	0.906^*^
RNFL temporal Quadrant	70.24 ± 12.33 [47–93]	73.30 ± 13.14 [45–101]	0.241^*^
Average sensitivity	29.74 ± 1.31^†^ [26.59–31.87]	29.3 ± 1.46 [23.9–32.03]	0.097^†^
VF superior division	30.05 ± 1.54 [27.04–32.91]	29.37 ± 1.56 [25.08–32.3]	*0.034* ^*^
VF inferior division	31.13 ± 1.43^†^ [27.28–32.91]	30.89 ± 1.37 [26.57–33.66]	0.211^†^
VF central division	27.59 ± 1.44 [23.28–31.14]	27.43 ± 1.83 [24–32.14]	0.406^†^
VF temporal división	30.19 ± 2.0 [25.67–33.33]	29.49^†^ ± 2.75 [13–33]	0.145^†^
VFI	99.16^†^ ± 0.85 [97–100]	98.97^†^ ± 0.983 [96–100]	0.361^†^
MD	0.04 ± 1.13 [(− 2.78) – 1.79]	−0.389 ± 1.2 [(− 3.75) – 1.65]	0.07^*^
PSD	1.52^†^ ± 0.3 [0.96–2.64]	1.61^†^ ± 0.67 [0.9–5.93]	0.955^†^

*IOP* intraocular pressure, *RNFL* retinal nerve fiber layer, *VF* visual field, *VFI* visual field index, *MD* mean deviation, *PSD* pattern standard deviation, *N* number of eyes

^*^T-student test (*p* value < 0.05). Normal distribution confirmed (Shapiro-Wilk)

^†^U Mann-Whitney test. No normal distribution confirmed (Shapiro-Wilk)

Table 2 shows the results of IOP and peripapillary RNFL thickness measurements by dividing OSAHS patients according to severity. IOP showed no differences between groups. Nasal quadrant of peripapillary RNFL thickness showed a difference between categories. Thus, nasal RNFL thickness was significantly lower in severe OSAHS than that in moderate OSAHS (*p* = 0.031, Mann-Whitney U test). Moreover, nasal RNFL thickness was thinner in severe OSAHS versus controls (*p* = 0.016, Mann-Whitney U test).

**Table 2:** Comparison of IOP and RNFL thickness measurements between controls, mild, moderate and severe OSAHS patients

			Normality S-W	Homogeneity Levene Test	ANOVA/Kruskall W	
	Severity	Average ± SD [Min-Max]	*p*-value	*p*-value	F	*p*-value
IOP	Control (*N* = 38)	15.1 ± 2.1 [10–19]	0.222	0.22	0.73	0.48^*^
	Mild-Moderate (*N* = 29)	15.6 ± 2.6 [11–22]	0.138			
	Severe (*N* = 34)	15.8 ± 3 [10–21]	0.135			
Average RNFL	Control (*N* = 38)	98.6 ± 10.9 [68.9–117.5]	0.617	0.78	2.13	0.12^*^
	Mild-Moderate (*N* = 29)	101.8 ± 10.4 [82.2–125.3]	0.651			
	Severe (*N* = 34)	96.3 ± 10.1 [76–118]	0.684			
RNFL superior quadrant	Control (*N* = 38)	119.9 ± 19.3 [69–159]	0.914	0.13	0.58	0.56^*^
	Mild-Moderate (*N* = 29)	124.5 ± 13.6 [85–160]	0.215			
	Severe (*N* = 34)	122 ± 18.4 [90–162]	0.695			
RNFL nasal quadrant	Control (*N* = 38)	80.1 ± 16.9 [52–126]	0.126	0.21	/	*0.029* ^*†*^
	Mild-Moderate (*N* = 29)	79.0 ± 17.0 [47–117]	0.533			
	Severe (*N* = 34)	71.3 ± 14.1 [49–115]	0.05			
RNFL inferior quadrant	Control (*N* = 38)	124.2 ± 13.7 [93–160]	0.829	0.07	1.92	0.15^*^
	Mild-Moderate (*N* = 29)	128.1 ± 19.9 [91–166]	0.688			
	Severe (*N* = 34)	120.2 ± 13.9 [92–163]	0.398			
RNFL temporal quadrant	Control (*N* = 38)	70.2 ± 12.3 [47–93]	0.410	0.91	1.34	0.27^*^
	Mild-Moderate (*N* = 29)	75.3 ± 12.6 [51–101]	0.910			
	Severe (*N* = 34)	71.6 ± 13.5 [45–100]	0.272			

*IOP* intraocular pressure, *RNFL* retinal nerve fiber layer, *S-W* Shapiro-Wilk, *Kruskall W* Kruskall Wallis, *N* number of eyes

^*^ANOVA test

^†^Kruskal Wallis (*p* value < 0.05)

Humphrey VF results showed no differences between controls and severity of OSAHS groups (Table 3). VF superior division showed a nonstatistical trend (0.08, ANOVA test). This difference was not statistically significant when we applied a multiple comparison test: *p* = 0.11 in controls versus mild-moderates; *p* = 0.295 in controls versus severes; and *p* = 0.834 in mild-moderates versus severes (Scheffé post hoc test).

**Table 3:** Comparison of Humphrey visual field sensitivities and indices between controls, mild, moderate and severe OSAHS patients

			Normality S-W	Homogeneity Test Levene	ANOVA / Kruskall W	
	Severity	Average ± SD [Min - Max]	*p*-value	*p*-value	F	*p*-value
Average sensitivity (dB)	Control (*N* = 38)	29.7 ± 1.3 [26.6–31.8]	0.04	0.61	/	0.2^†^
	Mild- Moderate (*N* = 29)	29.1 ± 1.6 [23.9–31.1]	0.4			
	Severe (*N* = 34)	29.4 ± 1.3 [26.0–32]	0.61			
VF superior division (dB)	Control (*N* = 38)	30.1 ± 1.5 [27–32.9]	0.158	0.83	2.49	0.08^*^
	Mild- Moderate (*N* = 29)	29.2 ± 1.6 [26.3–32.2]	0.7			
	Severe (*N* = 34)	29.5 ± 1.5 [25.1–32.3]	0.19			
VF inferior division (dB)	Control (*N* = 38)	31.1 ± 1.4 [27.3–33.3]	0.01	0.62	/	0.46^†^
	Mild- Moderate (*N* = 29)	30.9 ± 1.3 [26.6–32.9]	0.008			
	Severe (*N* = 34)	30.9 ± 1.4 [27.6–33.7]	0.91			
VF central division (dB)	Control (*N* = 38)	27.6 ± 1.4 [23.3–31.1]	0.46	0.11	0.73	0.49^*^
	Mild- Moderate (*N* = 29)	27.2 ± 1.6 [24.3–31.4]	0.49			
	Severe (*N* = 34)	27.6 ± 2.0 [24–32.1]	0.17			
VF temporal division (dB)	Control (*N* = 38)	30.2 ± 2 [25.7–33.3]	0.06	0.08	/	0.34^†^
	Mild- Moderate (*N* = 29)	29.7 ± 1.9 [26.3–33]	0.03			
	Severe (*N* = 34)	29.8 ± 1.7 [25.3–33]	0.33			
VFI (dB)	Control (*N* = 38)	99.2 ± 0.8 [97–100]	< 0.001	0.63	/	0.6^†^
	Mild- Moderate (*N* = 29)	98.9 ± 1.0 [96–100]	0.001			
	Severe (*N* = 34)	99.0 ± 0.9 [97–100]	< 0.001			
MD (dB)	Control (*N* = 38)	0.04 ± 1.1 [(−2.8) – 1.8]	0.08	0.92	1.66	0.19^*^
	Mild- Moderate (*N* = 29)	−0.4 ± 1.12 [(−2.5) – 1.6]	0.21			
	Severe (*N* = 34)	−0.4 ± 1.2 [(−3.8) - 1.7]	0.44			
PSD (dB)	Control (*N* = 38)	1.5 ± 0.3 [0.9–2.6]	0.01	0.1	/	0.97^†^
	Mild- Moderate (*N* = 29)	1.6 ± 0.9 [1.0–5.9]	0.002			
	Severe (*N* = 34)	1.6 ± 0.4 [0.9–2.8]	< 0.001			

*VF* visual field, *VFI* visual field index, *MD* mean deviation, *PSD* pattern standard deviation, *S-W* Shapiro-Wilk, *Kruskall W* Kruskall Wallis, *N* number of eyes

^*^ANOVA test

^†^Kruskal Wallis

A relationship between functional and structural ON parameters in both, OSAHS and controls, was not demonstrated. Table 4 shows linear, logarithmic, inverse, quadratic and cubic correlation models between linear parameters, as the thickness of peripapillary RNFL quadrants and parameters expressed in logarithmic scale such as VF sensitivities.

**Table 4:** Correlation models between structural and functional optic nerve variables in OSAHS patients

	OSAHS (*N* = 63)		CONTROLS (*N* = 38)		OSAHS (*N* = 63)		CONTROLS (*N* = 38)	
Regression model	R^2^	F Test (*p*-value)	R^2^	F Test (*p*-value)	R^2^	F Test (*p*-value)	R^2^	F Test (*p*-value)
	RNFL inferior quadrant (μm) – VF superior división (dB)				RNFL Superior quadrant (μm) – VF inferior división (dB)			
Lineal	0.042	0.106	0.04	0.226	0.054	0.068	0.002	0.777
Logarithmic	0.049	0.083	0.046	0.196	0.047	0.087	0.006	0.631
Inverse	0.054	0.066	0.052	0.169	0.04	0.117	0.014	0.484
Quadratic	0.076	0.095	0.067	0.294	0.071	0.11	0.054	0.376
Cubic	0.077	0.090	0.067	0.294	0.07	0.114	0.054	0.376
	RNFL nasal quadrant (μm) - VF temporal división (dB)				RNFL temporal quadrant (μm) - VF central división (dB)			
Lineal	0.014	0.362	0.006	0.644	0.004	0.642	0.001	0.831
Logarithmic	0.008	0.48	0.01	0.547	0.005	0.580	0.003	0.76
Inverse	0.004	0.622	0.015	0.468	0.007	0.518	0.004	0.709
Quadratic	0.045	0.25	0.048	0.421	0.012	0.691	0.045	0.447
Cubic	0.055	0.339	0.05	0.62	0.012	0.691	0.052	0.393
	Average RNFL (μm) – MD (dB)				Average RNFL (μm) - Average sensitivity (dB)			
Lineal	0.005	0,585	0.011	0.531	0.007	0.514	0.008	0.584
Logarithmic	/	/	/	/	0.008	0.489	0.01	0.557
Inverse	0.017	0.309	0.001	0.861	0.009	0.469	0.011	0.533
Quadratic	0.046	0.241	0.044	0.453	0.014	0.663	0.074	0.261
Cubic	0.061	0.291	0.046	0.658	0.014	0.66	0.075	0.255

*RNFL* Retinal nerve fiber layer, *MD* mean deviation, *VF* visual field. *N* number of eyes

**Fig. 2:**
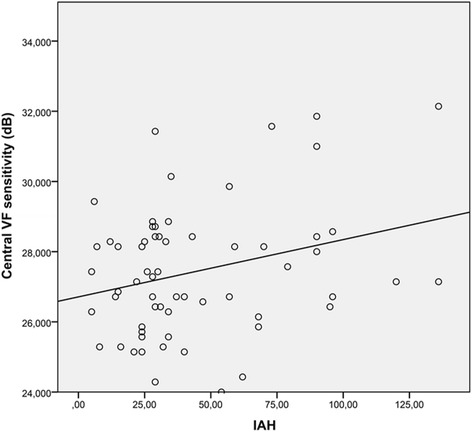
Pearson’s correlation coefficient (0.284, *p* = 0.024) between central VF sensitivity and AHI

Only the central VF sensitivity of the SAP revealed a poor correlation with the AHI, with a Pearson’s correlation coefficient of 0.284 (*p* = 0.024, Fig. 2).

## Discussion

The low oxygen levels during sleep lead to the adrenergic system activation, inflammation and procoagulant mechanisms prompting to endothelial dysfunction, oxidative stress and metabolic deregulation [23]. This vascular phenomenon may compromise ON perfusion and oxygenation, ultimately leading to optic neuropathy [24, 25]. Several authors, including our own group, have published RNFL thickness variations in individuals with sleep apnea [11–16], which could be a consequence of vascular and gasometric alterations triggered by OSAHS.

Peripapillary RNFL thickness reflects neuronal axons, and would allow quantification of ganglion cell axonal loss [26]. Moreover, VF values estimates functional loss of the whole visual pathway, from retina to cortex [27]. VF maps show a correspondence with neuronal distribution of RNFL in the ON head, as Garway et al. demonstrated [17]. Therefore, theoretically, a correlation between OCT and VF parameters could be found. If this correspondence appears, both OCT and VF could be potentially used as biomarkers for neuronal degeneration in OSAHS patients.

Sensitivity of superior VF division was significantly lower in OSAHS patients than that in controls. This finding is not consistent with a significant change of inferior peripapillary RNFL thickness, which corresponds topographically with superior VF division and would justify the functional decline. Subtle edema secondary to vascular deregulation and increase in cerebrospinal fluid pressure [13, 28, 29] could explain the VF alterations found in our patients, and the fact that reduction in sensitivity is not associated with a decrease in RNFL thickness. As we previously suggested [14], it is possible that analysing all ranges of OSAHS severity together imply masking certain ON structural alterations. Theoretically, a first stage of edema and inflammation in mild and intermediate stages of the respiratory disease would precede an atrophy phase. We should, therefore, think that the biggest differences will be found in the most serious cases, where the decline of the RNFL will be triggered by longer exposure to hypoxia, and will be logical find higher degrees of atrophy. This fact is reflected in nasal quadrant RNFL thickness, where we found differences between severe OSAHS and the other groups. Another possible theory explaining the functional loss without structural alteration is the association of OSAHS with floppy eyelid syndrome [30–32]. A fatigue-related ptosis could reduce superior visual field acting as an artefact, as the ‘normal’ OCT inferior RNFL thickness confirms.

On the other hand, VF parameters showed no differences in the two by two comparisons between OSAHS groups and controls. This result contrasts with those obtained by other authors. Thus, Huseyinoglu et al. [13] found higher PSD and lower MD values in OSAHS patients comparing to controls. Xin et al. and Tsang et al. [29, 33] found affected values of MD and PSD in OSAHS comparing with controls. Nevertheless, they did not exclude any case of glaucoma and that could generate a bias in their results. Ferrandez et al. [34] found a generalized decrease in retinal sensitivity without focal defects in OSAHS. Furthermore, they reported changes in MD, PSD and VFI values with worse scores in patients with apnea. Disparities between their results and ours could be justified by different examination strategies used. We have chosen a shorter VF strategy to explore the patients since we know that “fatigue effect” could modify VF outcomes [33, 35] and is more prevalent in OSAHS patients [36–38], even when the SAP reliability criteria were kept within normal limits. We could observe a considerable difficulty to some OSAHS patients to maintain the concentration for 4 to 8 min for VF completion. Because of this, we decided to perform SITA-fast strategies in the present study, in spite of this shorter algorithm is known to improve the mean defect and to be less precise compared to SITA standard [39]. It is likely that hypersomnia, which is one of the crucial symptoms of the disease, could act as a confounding factor during VF examination, obtaining lower sensitivity rates comparing to controls, especially in longer examinations.

Regarding structure-function analysis, the absence of a common scale for both measurements, logarithmic on one side (decibels) and decimal on the other one (microns or percentages), makes it difficult to draw a parallel relationship between both examinations. There is a controversy regarding the type of association between structural and functional variables. Thus, various publications assume a linear correlation when both parameters are expressed in a linear scale, and an exponential or curvilinear relationship when one of the variables is linear whereas the other one is expressed in a logarithmic scale [40, 41]. Conversely, other authors hardly found any differences between structure-function correlations when the latter was expressed in logarithmic or linear scale [42, 43]. Based on this, our research analyses the association applying linear and non-linear models.

Special interest would have the correlation between the nasal quadrant of RNFL thickness and temporal VF division, since we found statistically significant differences for the structural parameter. Relationship between RNFL thickness quantified with Stratus OCT and VF MD was better fitted with second-order polynomials than with a linear model in patients with glaucoma [41]. These regression models describe a curvilinear relationship, suggesting that progression of VF loss, when it is expressed in MD, increases during the course of the disease. This idea is compatible with the concept of “functional reserve”, i.e. there must be a significant structural damage to bring up a functional representation of the same in the VF [19, 44–47]. Therefore, the absence of correlation found in the present study may be a consequence of the perimetric exclusion criteria, by which all eyes with suggestive alterations of glaucomatous neuropathy were excluded from the analysis.

One of the strengths of our study is that we just included patients with normal IOP, normal gonioscopy, and no perimetric evidence of glaucomatous neuropathy, in order to assess a hypothetical reduction in retinal sensitivity produced exclusively by the respiratory disorder. In this way, we avoid biased results by the inclusion of glaucomatous patients, whose incidence, as we have already mentioned, is higher in patients suffering from apnea [7–10]. The main limitation of our study include the relatively small sample size, mainly when OSAHS were divided according to severity. Groups with larger number of patients could have shown some correlation between variables or more consistent conclusions.

## Conclusions

In conclusion, due to the lack of association between variables and no significant differences in VF variables when dividing OSAHS patients, we cannot state that perimetry is a useful diagnostic tool to demonstrate functional deterioration in OSAHS. Moreover, future research should analyze the results obtained in patients with OSAHS prior and after effective treatment, to establish the real VF involvement in the apneic disorder.

## Abbreviations

AHI: Apnea–hypopnea index
BCVA: Best-corrected visual acuity
MD: Mean deviation
MS: Mean sensitivity
OCT: Optical coherence tomography
ON: Optic nerve
OSAHS: Obstructive sleep apnea hypopnea syndrome
PSD: Pattern standard deviation
RNFL: Retinal nerve fiber layer
SAP: Standard automated perimetry
VF: Visual field
